# Abundances of placental imprinted genes *CDKN1C*, *PHLDA2* and *IGF-2* are related to low birth weight and early catch-up growth in full-term infants born small for gestational age

**DOI:** 10.1371/journal.pone.0218278

**Published:** 2019-06-13

**Authors:** Yan Xing, Huiqiang Liu, Yunpu Cui, Xinli Wang, Xiaomei Tong

**Affiliations:** Department of Pediatrics, Peking University Third Hospital, Beijing, China; Centre Hospitalier Universitaire Vaudois, FRANCE

## Abstract

Children born small for gestational age (SGA) generally have a catch-up growth and rapid weight gain in the first years of life, which is a high risk of insulin resistance and cardiovascular diseases later in life. It was reported that the level of imprinted genes *IGF-2*, *CDKN1C* and *PHLDA2* regulates placental growth. We assessed these imprinted genes expression levels in placental tissue and their influences on catch-up growth of full-term SGA infants. The protein and mRNA levels of placental *CDKN1C*, *PHLDA2* and *IGF-2* were analyzed in 29 full-term SGA and 29 full-term infants born appropriate for gestational age (AGA) using quantitative RT-PCR and Western blot assay, respectively. Catch-up growth was indicated by increased standard deviation score (ΔSDS) of weight at 1, 3 and 6 months relative to birth weight (BW). Correlations between indicated variables were evaluated using Pearson correlation coefficient analysis. Compared to AGA infants, *CDKN1C* and *PHLDA2* levels were significantly increased, whereas *IGF-2* was significantly reduced in SGA infants. The value of ΔSDS was significantly higher in SGA than that in AGA infants. For SGA status, Pearson analysis shows i) a negative correlation of *CDKN1C* and *PHLDA2* abundances with BW, and a positive correlation of *IGF-2* with BW, ii) no correlation between the three imprinted gene abundances and placental weight (PW), and between PW and BW, iii) a positive correlation of *PHLDA2* abundance with *CDKN1C*, and iv) a positive correlation of *CDKN1C* and *PHLDA2* abundances with ΔSDS, and a negative correlation of *IGF-2* with ΔSDS at 1, 3 and 6 months. Taken together, increased *CDKN1C* and *PHLDA2* and reduced *IGF-2* abundances in placental tissue were related to BW and early period catch-up growth in full-term SGA infants. Placental *CDKN1C*, *PHLDA2* and *IGF-2* level monitoring may be useful for predicting and preventing the development of SGA.

## Introduction

Small for gestational age (SGA) is generally defined as low birth weight (BW) at 10^th^ percentile or at less than -2 standard deviations from the mean [1,2]. Little is known about the exact underlying mechanism by which SGA births occur. It has been reported that several factors are related to the development of SGA [1,2]. The lack of nutritional supply to the fetus is regarded as one of the major causes of reduced fetal growth [3]. Alterations of the expressions of specific imprinted genes are related to appropriate fetal and placental growth [4].

Genomic imprinting is an epigenetic phenomenon that causes genes to be expressed in a parent-of-origin-specific manner and predominately active in the placenta during fetal development [4,5]. As of 2014, there are about 150 imprinted genes known in mouse and about half of those in human [6]. Insulin-like growth factors (IGFs) have a major role in promoting embryonic and fetal growth as well as growth during infancy and childhood. In accordance with this role, abnormalities of IGFs level were found in the SGA children. It was reported that mean serum levels of IGF-1 and IGF-binding protein 3 in the SGA infants at birth were significantly lower than those in the appropriate for gestational age (AGA) births [7,8]. The *IGF-2* allele only inherited from the father (paternal) is expressed in humans. The plasma levels of IGF-2 proteins were lower in the term SGA infants after birth compared to the term AGA infants [9]. Similarly, the mRNA levels of IGF-2 in chorionic villus in the SGA neonates were also significantly lower than those in the AGA neonates. Moreover, it has been found that the transcript level of IGF-2 was positively correlated with BW [10]. The imprinted gene pleckstrin homology-like domain family A member 2 (*PHLDA2*) is maternally expressed in both humans and mice. In *PHLDA2* knockout mice, a significant increase of placental size was detected during mid to late gestation [11]. Increased placental expression of *PHLDA2* was found in the SGA infants, and negatively associated with BW [12,13]. Cyclin dependent kinase inhibitor 1C (*CDKN1C*), another crucial imprinted gene, plays an important role in regulation of embryonic growth [14,15]. *CDKN1C* and *IGF-2* were significantly upregulated in the assisted reproductive technology conceived placentas (ARTCP), and the mean BW of the singletons from ARTCP was obviously lower than that of naturally conceived ones. Increased percentage of SGA births was also reported in the ARTCP [16]. However, the alterations and the roles of these imprinted genes are not consistent in SGA, particularly at old ages. It was reported that the mRNA level of chorionic villus PHLDA2 in early gestation did not have effect on BW [10]. A persistent downregulation of the IGF-1 levels was reported in the SGA children [17], whereas upregulated IGF-1 level was found in the SGA children with catch-up growth [7,8].

Compared to AGA births, SGA infants and/or children generally undergo catch-up growth and rapid weight gain in the first years of life. Some studies show that SGA children with rapid catch-up growth tend to develop metabolic problems such as insulin resistance and cardiovascular diseases [1,2]. Although the link between these imprinted genes and fetal growth has been described in humans and in animal species, the investigations about the association of their expression levels with catch-up growth remains few in SGA.

In this study, we assessed the levels of three imprinted genes *CDKN1C*, *PHLDA2* and *IGF-2* in placental tissue and analyzed their influences on catch-up growth in full-term SGA infants. Our findings show that increased *CDKN1C* and *PHLDA2* and reduced *IGF-2* abundances in placental tissue were related to BW reduction and early period catch-up growth in the SGA infants.

## Materials and methods

### Subjects

This study was approved by the Medical Ethics Committee of the Peking University Third Hospital (Protocol number: IRB00006761-2012005). Written informed consent was obtained from the parents of infants prior to the study participation.

This study was carried out at the NICU of the Peking University Third Hospital (Beijing, China). A total of 58 infants born at the hospital between June 2014 and June 2016 were recruited, including 29 SGA and 29 AGA. Gestational age was estimated from the mother’s last menstrual period and was confirmed through fetal ultrasound measurements. SGA was defined as the BW and birth length being below the 10th percentile of this reference. We excluded infants with major congenital abnormalities, severe asphyxia or infection.

General information was obtained, including mother’s age, height, weight, gestational age, placental weight (PW), delivery way, and delivery times; father’s age, weight, and height; infant’s birth length, weight at birth as well as at 1, 3 and 6 months (Table 1).

**Table 1:** General characteristic of subjects.

	SGA	AGA	*t/χ*^*2*^	*p*
*n* (male/female)	29 (16/13)	29 (15/14)		
Gestational age (weeks)	38.7 ± 1.1	38.6 ± 1.2	0.344	*0*.*732*
Infants weight (g)				
At birth	2491.4 ± 204.8	3418.6 ± 331.8	-12.820	*<0*.001*
1 month	3292.4 ± 219.0	4195.9 ± 390.7	-10.863	*<0*.001*
3 months	5291.4 ± 331.1	6383.8 ± 530.1	-9.413	*<0*.001*
6 months	7601.0 ± 521.3	8180.7 ± 499.5	-4.323	*<0*.001*
Birth length (cm)	45.2 ± 1.3	50.1 ± 1.8	-11.746	*<0*.001*
Placental weight (g)	471.5 ± 27.9	564.5 ± 35.3	-11.115	*<0*.001*
Mother’s age (years)	29.5 ± 4.2	28.4 ± 2.4	1.200	*0*.*235*
Mother’s weight before Pregnancy (kg)	54.0 ± 2.9	53.3 ± 2.2	1.086	*0*.*282*
Mother’s increased weight (kg)	14.7 ± 1.2	15.2 ± 1.1	-1.944	*0*.*057*
Mother’s height (cm)	162.1 ± 4.8	162.5 ± 4.3	-0.316	*0*.*753*
Delivery way				
Natural	15	14	0.279	*0*.*792*
C. section	17	12		
Delivery times				
Single	25	23	0.483	*0*.*730*
Multiple	4	6		
Father’s age (years)	29.8 ± 2.0	28.7 ± 2.3	1.946	*0*.*057*
Father’s weight (kg)	70.9 ± 5.6	71.6 ± 4.6	-0.462	*0*.*646*
Father’s height (cm)	171.7 ± 4.2	172.5 ± 3.4	-0.793	*0*.*431*

Data are presented as means ± standard deviations or number of subjects. AGA: appropriate for gestational-age. SGA: small for gestational age.

*p < 0.*05*, SGA versus AGA.

According to nutritional protocol, feeding was typically initiated within the first 8 h after birth (20 mL/kg divided over 8 feeds, every 3 h per day); the type of feeding depended on the mother’s willingness and ability to provide breast milk. When breast milk was unavailable, infants received Enfamil Premium Infant Formula (Mead Johns & Company, LLC., Evansville, IN, USA).

### Reverse transcription-quantitative polymerase chain reaction (RT-qPCR)

Total RNA was extracted from 50 mg placental tissue per sample with Trizol reagent (cat. no. 15596026; Thermo Fisher Scientific, Inc., Wilmington, DE, USA) followed by DNase I (TakaRa, Otsu Shiga, Japan) treatment. 1.5 μg of RNA was reversely transcribed to cDNA in a 25 μl reaction system with the First-Strand cDNA Synthesis kit (cat. no. 11483188001; Sigma-Aldrich). qPCR was performed with the SYBR Green PCR Master Mix (cat. no. 1725270; Bio-Rad Laboratories, Inc.), including 2.0 μl of cDNA and 0.25 μM of specific primer pairs (Table 2). PCR program was run on a PE5700 Real-Time PCR system (Bio-Rad Laboratories, Inc., Hercules, CA, USA): initial denaturation at 95˚C for 30 sec, 40 cycles of 30 sec at 95˚C and 30 sec at annealing temperature. The mRNA expression levels of the *CDKN1C*, *PHLDA2* and *IGF-2* were calculated using the 2^-ΔΔCt^ method, and was normalized by the geometric mean of the ΔCt of the housekeeping gene *β-actin* and glyceraldehyde-3-phosphate dehydrogenase (*GAPDH)*.

**Table 2:** Primer sequences employed for reverse transcription-quantitative polymerase chain reaction.

Gene	Primers	Annealing Tm	Product size
*CDKN1C*	F: 5’-atgtccgacgcgtccct-3’	55°C	192bp
	R: 5’- gtcgtaatcccagcggttct-3’		
*IGF-2*	F: 5’- cccctccgaccgtgct-3’	56°C	164bp
	R: 5’—tcatattggaagaacttgccca-3’		
*PHLDA2*	F: 5’- gagcgcacgggcaagta-3’	57°C	168bp
	R: 5’—cagcggaagtcgatctcctt-3’		
*β-actin*	F: 5’- agccatgtacgtagccatcca-3’	56°C	142bp
	R: 5’- tctccggagtccatcacaatg-3’		
*GAPDH*	F: 5’-gtctcctctgacttcaacagcg-3’	58°C	131bp
	R: 5’-accaccctgttgctgtagccaa-3’		

### Western blot

Total protein was extracted from 100 mg placental tissue using radioimmunoprecipitation assay buffer (25 mmol·L^-1^ Tris-HCl pH 7.4, 150 mmol·L^-1^ NaCl, 1% Nonidet-40, 0.1% SDS, 0.5% sodium deoxycholate) supplemented with fresh protease inhibitor (cat. no. A32965; Thermo Fisher Scientific, Inc.), and quantified using the BCA Protein Assay Kit (cat. no. 23225; Thermo Fisher Scientific, Inc.). A total of 100 μg protein was separated using 12.5% SDS-PAGE, and then transferred to the Nitrocellulose Membranes (cat. no. GERPN82D; Sigma-Aldrich). The membrane was blocked for 1 h in 5% BSA prepared in Tris-buffered saline containing 0.05% Tween-20 (TBST), and then incubated for overnight at 4°C with the primary antibodies: rabbit anti-CDKNC1 monoclonal antibody (1:500; cat. no. HPA002924; Sigma-Aldrich), rabbit anti-PHLDA2 polyclonal antibody (1:1,000; cat. no. PA5-76870; Invitrogen; Thermo Fisher Scientific, Inc.), rabbit anti-IGF-2 polyclonal antibody (1:200; cat. no. ab9574; Abcam), and rabbit anti-β-actin monoclonal antibody (1: 2,000; cat. no. SAB4301137; Sigma-Aldrich). After 3 washes with TBST, the membranes were incubated with horseradish peroxidase conjugated goat anti-rabbit IgG (1:10,000; cat. no. G-21234; Invitrogen; Thermo Fisher Scientific, Inc.) for 1 h, followed by developing with the Enhanced Chemiluminescence Western Blotting Substrate (cat. no. W1001; Promega, Madison, WI, USA). The intensity of the specific bands was quantified using ImageJ software (version 1.51s; National Institute of Health, Bethesda, MD, USA).

### Statistical analysis

The data are presented as means ± standard deviations (SD) or number of subjects. The unpaired *t*-test with the Mann-Whitney test was used to compare differences between two groups. Multiple unpaired *t-tests* with the Holm-Sidak correction were used to compare differences between two groups at multiple time points. Correlations between the indicated variables were evaluated using Pearson correlation coefficient analysis. The *p* values ≤ 0.05 were considered statistically significant, and all statistical analyses were performed using SPSS11.0 software (IBM Corporation, Armonk, NY, USA).

## Results

### Comparisons of general information between SGA and AGA infants

The clinical characteristics and anthropometric indices of the SGA and AGA infants are summarized (Table 1**)**. There were no significant differences in the gestational age, and mother’s age, weight, height and delivery way as well as father’s age, weight and height between the SGA and the AGA infants. In this study, both SGA and AGA infants were full term infants with around 38.7 and 38.6 weeks of gestational age. The SGA infants had significantly (*p < 0*.*001*) reduced BW and birth length as well as reduced weight at 1, 3 and 6 months after birth in comparison with the AGA infants. In addition, PW exhibited a significant (*p < 0*.*001*) reduction in the SGA infants compared to that in the AGA.

### Comparisons of placental tissue imprinted genes expression levels between SGA and AGA infants

**Fig 1:**
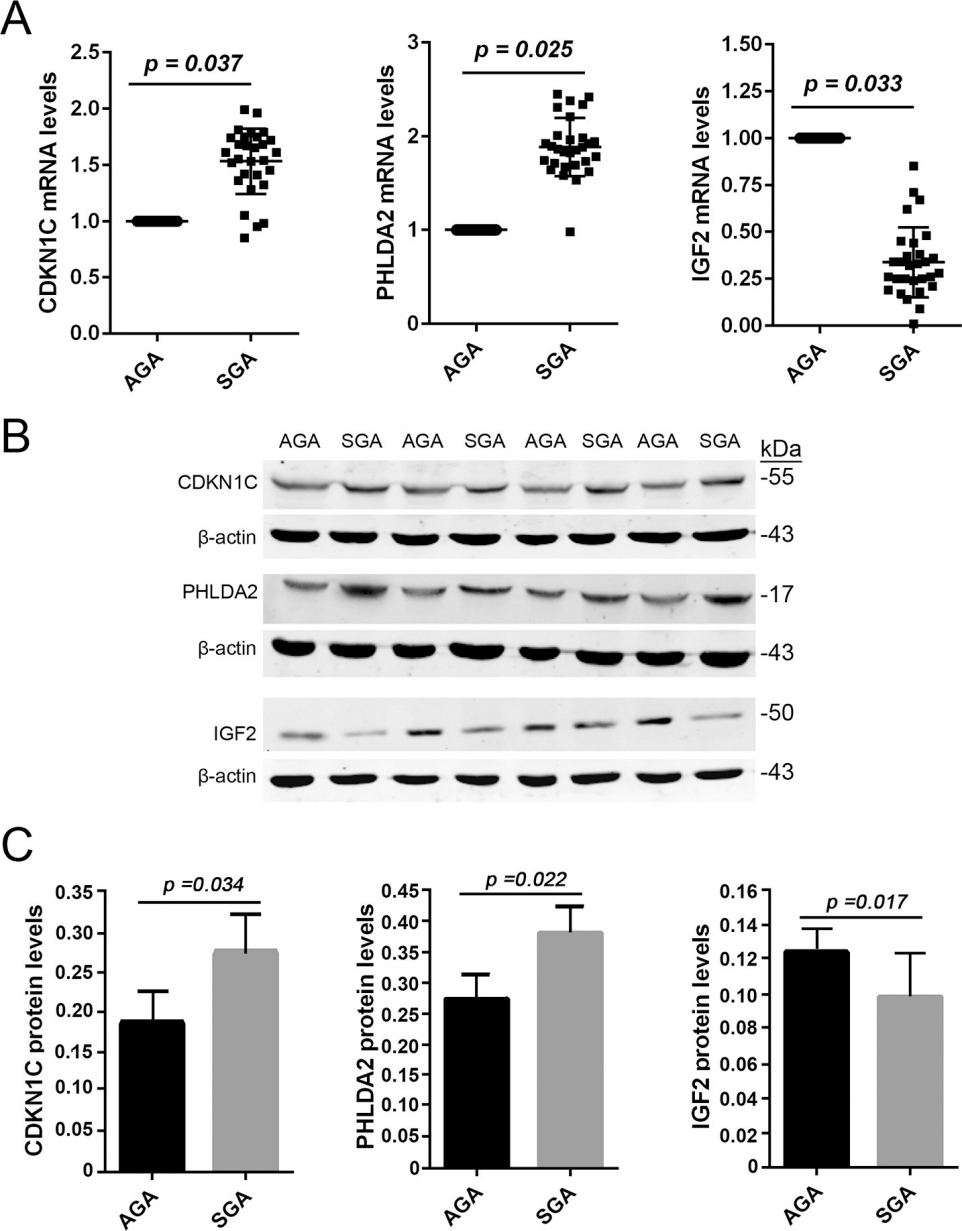
Expression levels of imprinted genes in placenta tissue. The expression levels of imprinted genes in placenta tissue were compared between the SGA and AGA infants. A. Total RNA was extracted from placental tissue, and real time qPCR was performed to measure the mRNA levels of *CDKN1C*, *PHLDA2* and *IGF-2*. B. Western blot assay was used to evaluate the protein levels of placental *CDKN1C*, *PHLDA2* and *IGF-2*. *Data are presented as mean ± SD*. *n = 29*. *p < 0.*05*, *SGA vs AGA*. *SGA*: *small for gestational age*. *AGA*: *appropriate for gestational age*.

It has been reported that the expression levels of specific imprinted genes might be related to fetal and placental growth [5]. In this study, we evaluated and compared the alterations of the expression levels of the placental imprinted genes *CDKN1C*, *PHLDA2* and *IGF-2* between the AGA and SGA infants. Quantitative RT-PCR results show that the mRNA levels of *CDKN1C* and *PHLDA2* was significantly (*p < 0*.*05*) higher, while those of *IGF-2* were significantly (*p < 0*.*05*) lower in the SGA infants than that in the AGA infants (Fig 1A). At protein levels, similar results were obtained from Western blot assay (Fig 1B). These data suggest that the abundances of the three imprinted genes were altered and may involve the development of SGA infants.

### Correlation analysis of the protein levels of imprinted genes with BW and PW

**Fig 2:**
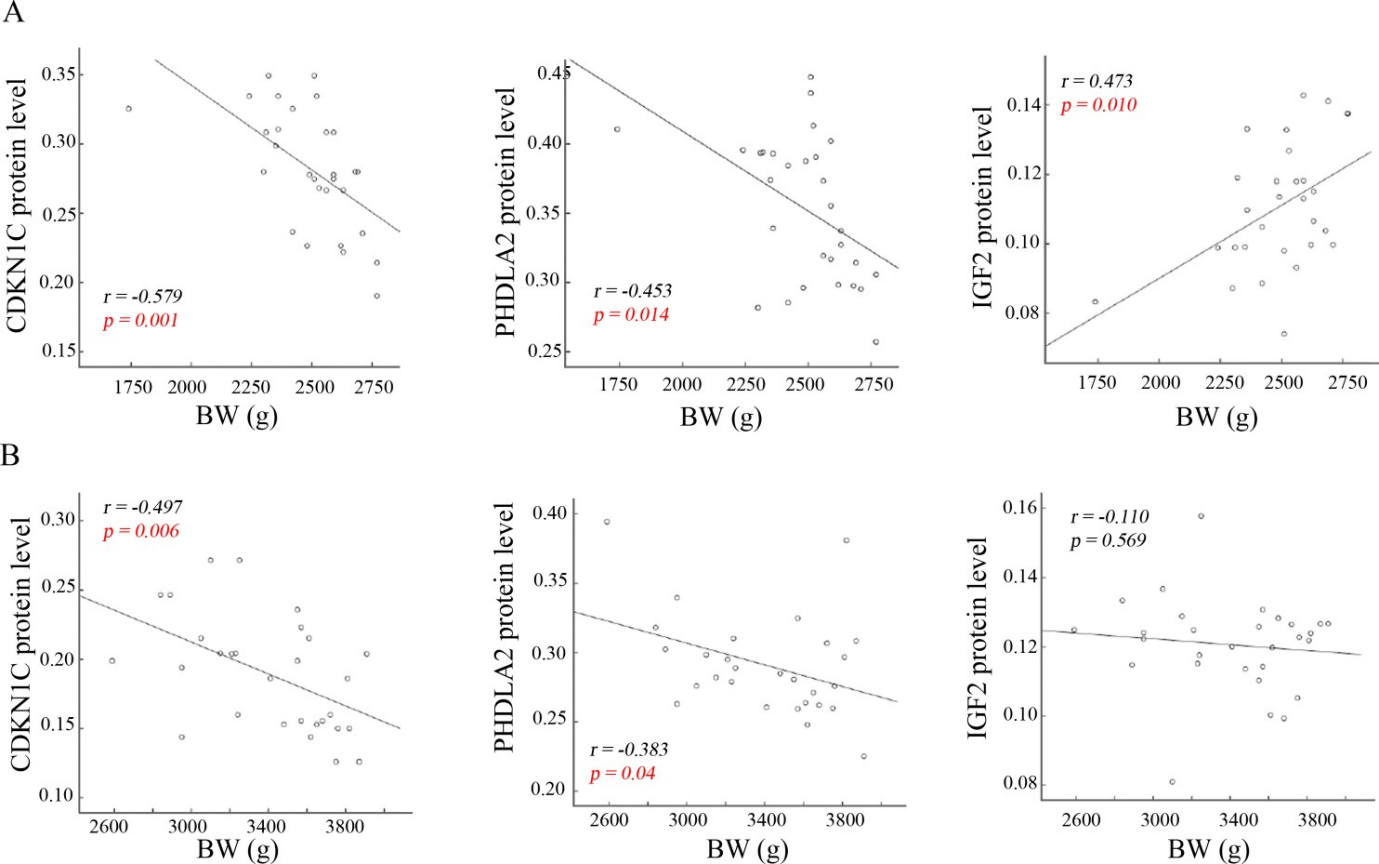
Association analysis of the protein levels of placental imprinted genes with birth weight. Association of the protein levels of placental imprinted genes with birth weight (BW, g) was performed in the SGA and AGA infants. A. In the SGA infants, *CDKN1C* and *PHDL2* protein levels are negatively associated with BW, whereas *IGF-2* protein levels show positive association with BW. B. In the AGA infants, *CDKN1C* and *PHDL2* protein levels are negatively associated with BW, whereas *IGF-2* protein levels do not show association with BW.

**Fig 3:**
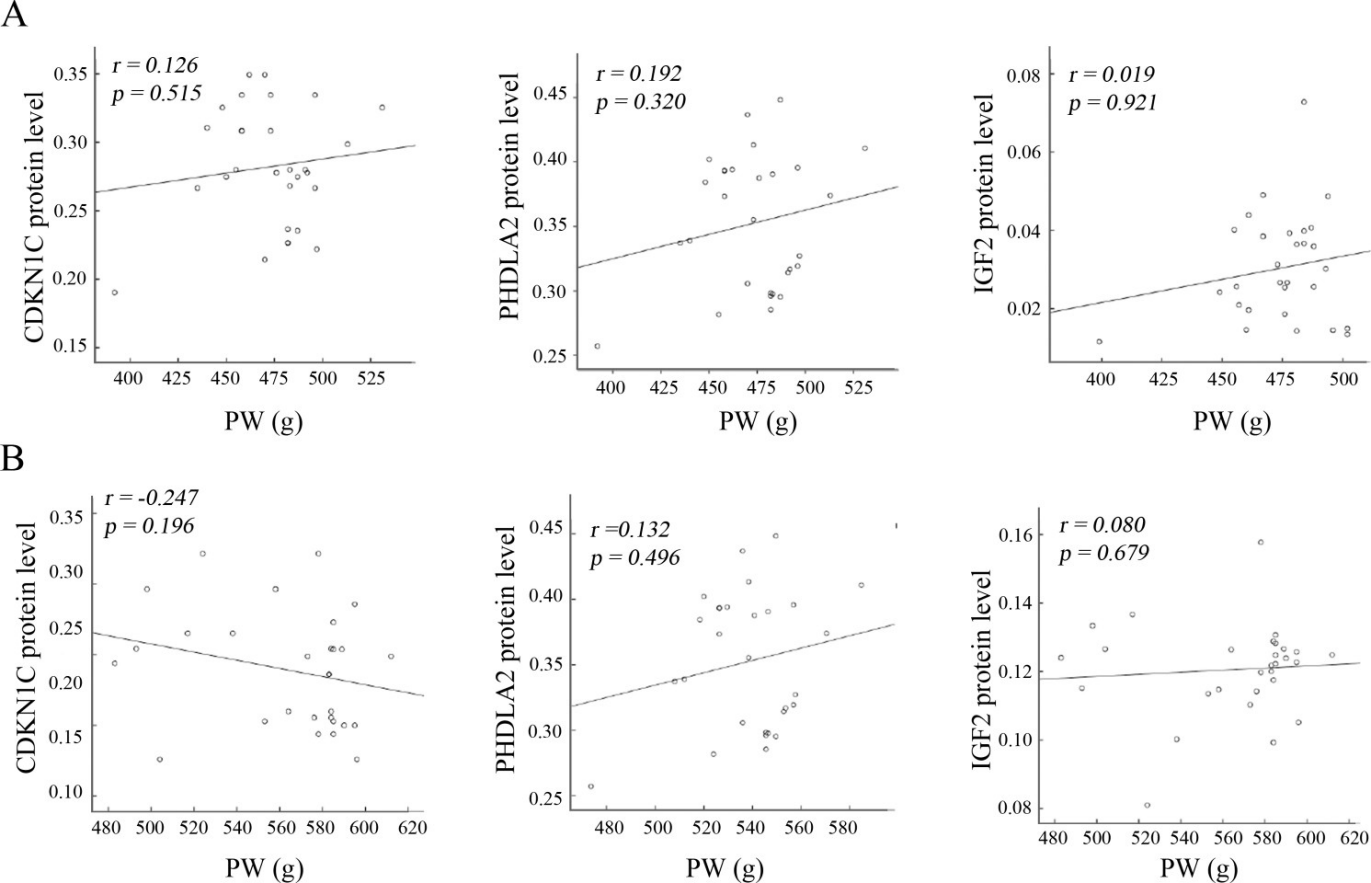
Association analysis of the protein levels of placental imprinted genes with placenta weight. Association of the protein levels of placental imprinted genes with placenta weight (PW, g) was performed in the SGA (A) and AGA (B) infants. Pearson’s correlation analysis does not show association of placenta imprinted genes’ protein levels with PW.

**Fig 4:**
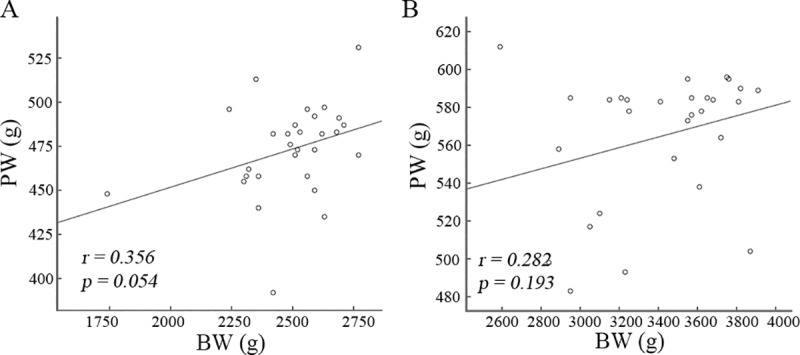
Association analysis of placenta weight with birth weight. Association of placenta weight (PW, g) with birth weight (BW, g) was performed in the SGA (A) and AGA (B) infants. Pearson’s correlation analysis does not show association of PW with BW.

BW and PW shows significant reduction in the SGA infants compared to the AGA (Table 1). Alterations of the expression levels of placental imprinted genes levels were revealed in the SGA infants (Fig 1). Therefore, we wanted to know if the protein abundances of the three imprinted genes have correlation with reduced BW and PW. Pearson’s coefficient analysis shows a significant (*p < 0*.*05*) negative correlation of *CDKN1C* and *PHLDA2* protein levels with BW both in the SGA and AGA infants. Moreover, a higher correlation of *CDKN1C* (*r*: -0.579 in SGA vs -0.497 in AGA) and *PHLDA2* (*r*: -0.453 in SGA vs -0.383 in AGA) with BW was observed in the SGA infants compared to the AGA (Fig 2). Of note, a positive association of *INF-2* protein levels with BW was only revealed in the SGA infants (*r = 0*.*473*, *p = 0*.*010*), but not in the AGA infants (*r = -0*.*110*, *p = 0*.*569*). Nevertheless, Pearson’s analysis does not show significant correlation (*p > 0*.*05*) of *CDKN1C*, *PHLDA2* and *INF-2* protein levels with PW both in the SGA and AGA infants (Fig 3). These data imply the importance of these imprinted genes in fetal growth, but not in placental weight. To address if reduced BW is caused by decreased PW, we analyzed the correlation of PW with BW, showing that there was no significant correlation between PW and BW both in the SGA and AGA infants (Fig 4).

### Positive correlation of *PHLDA2* with *CDKN1C* protein levels in the SGA infants

**Fig 5:**
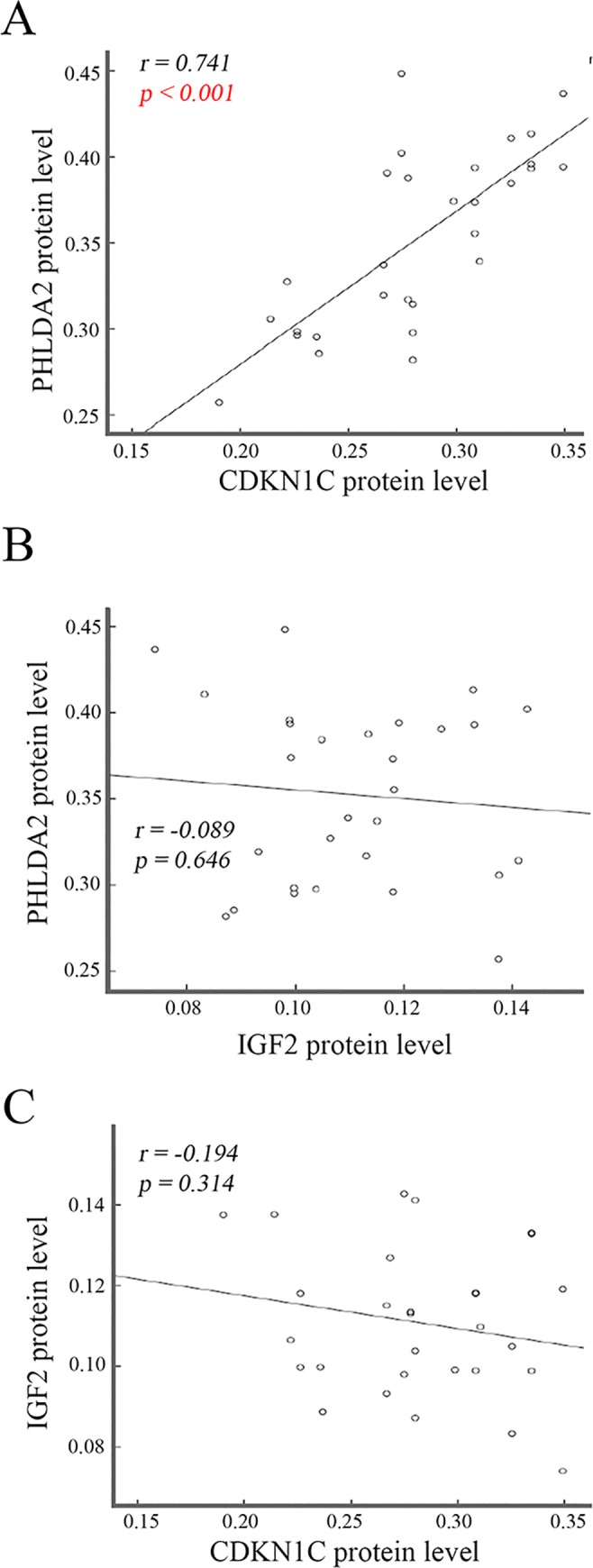
Association analysis of protein levels among placenta imprinted genes in the SGA infants. Association analysis was performed among placenta imprinted genes protein levels in SGA infants, showing a positive correlation of *PHLDA2* with *CDKN1C* (A) and no correlation of *PHLDA2* with *IGF-2* (B) as well as *IGF-2* with *CDKN1C* (C).

Imprinted genes levels were correlated with BW in SGA infants. We thus performed Pearson’s coefficient analysis between these imprinted genes protein levels. A significant positive association (*r = 0*.*741*, *p < 0*.*001*) of *PHLDA2* with *CDKN1C* was revealed (Fig 5A). We did not find significant association between *PHLDA2* and *IGF-2* as well as *IGF-2* and *CDKN1C* (Fig 5B and 5C).

### Comparison of catch-up growth between the SGA and AGA infants

**Fig 6:**
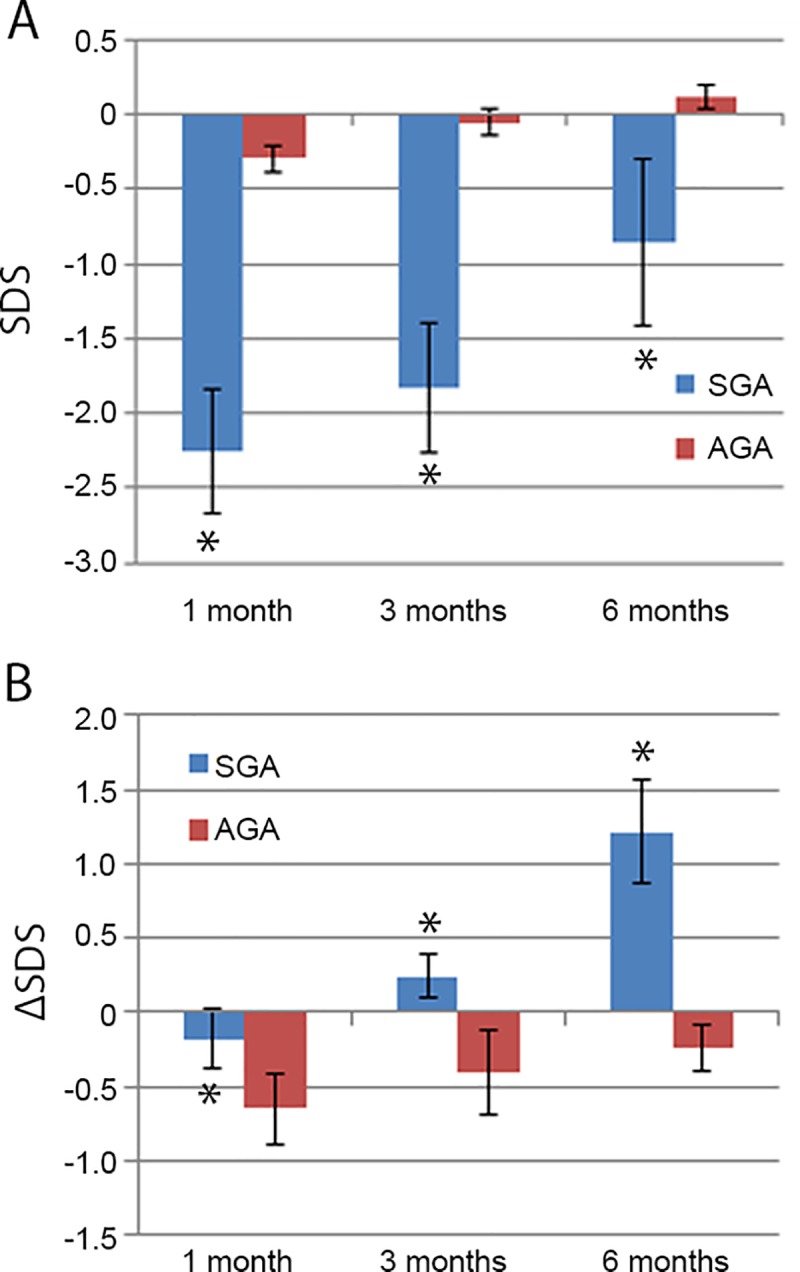
Comparison of catch-up growth between the SGA and AGA infants. Standard deviation score (SDS) of body weight was calculated as described in the Materials and Methods section, and the ΔSDS was used to indicate the weight gain and catch-up growth. A. Body weight of the SDS was compared at 1, 3 and 6 months after birth between the SGA and AGA infants. The values of the SDS in the SGA infants are significantly lower than that in the AGA infants. B. Weight gain and catch-up growth was compared using the ΔSDS at 1, 3 and 6 months after birth between the SGA and AGA infants. The values of the ΔSDS in the SGA infants are significantly higher than that in the AGA infants. *Data are presented as mean ± SD*. *n = 29*. *p < 0.*05*, *SGA vs AGA*. *SGA*: *small for gestational age*. *AGA*: *appropriate for gestational age*.

Standard deviation score (SDS) of the weight was calculated: SDS = (body weight—mean of body weight of the control group)/SD of the control group’s body weight, and the ΔSDS was calculated as the increased SDS at 1, 3 and 6 months after birth relative to that at birth. Here, the values of the ΔSDS represent the degree of weight gain and catch-up growth. The values of the SDS were compared at 1, 3 and 6 months after birth between the SGA and AGA infants, respectively. In the SGA infants, the values of the SDS were reduced significantly (*p < 0*.*01*) at 1, 3 and 6 months, whereas the values of the ΔSDS were increased significantly (*p < 0*.*05*) at all three observed time points, compared to the AGA infants (Fig 6). These data suggest a rapid catch-up growth since 1 month after birth in the SGA infants relative to the AGA infants although the weights of the SGA infants were still lower than that of the AGA infants.

**Fig 7:**
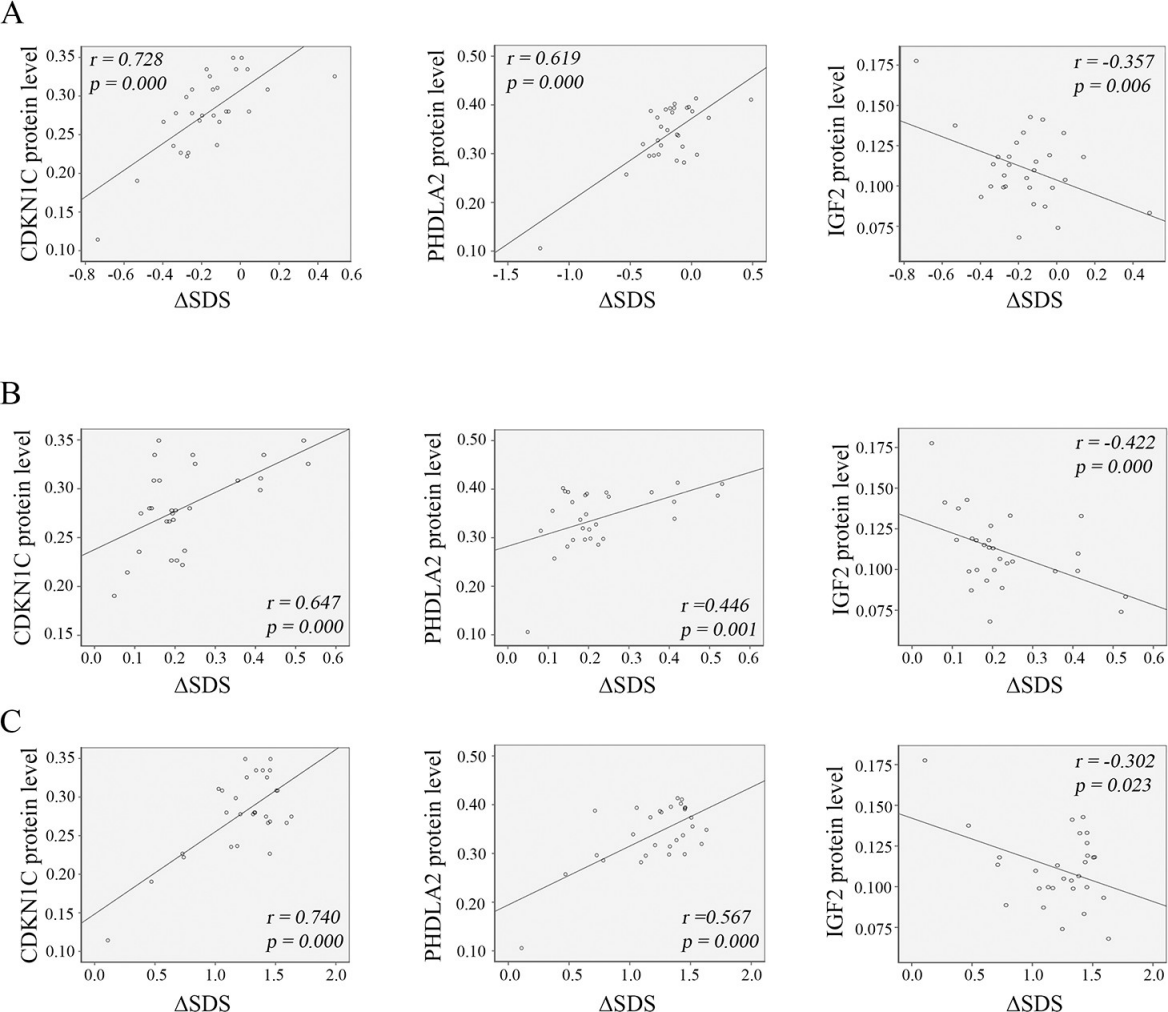
Association analysis of the protein levels of imprint genes with the ΔSDS in the SGA infants. Standard deviation score (SDS) of body weight was calculated as described in the Materials and Methods section, and the ΔSDS was used to indicate the weight gain at 1 (A), 3 (B) and 6 (C) months after birth relative to the birth weight. Pearson correlation analysis was performed to show the association of the protein levels of these imprinted genes with the ΔSDS.

We also analyzed the correlation of the protein levels of these imprinted genes with the ΔSDS in the SGA infants (Fig 7). Our data show that the *CDKN1C* and *PHLDA2* protein levels were positively (*p < 0*.*05*) correlated with the ΔSDS, whereas the *IGF-2* protein levels were negatively (*p < 0*.*05*) correlated with the ΔSDS at 1, 3 and 6 months after birth in the SGA infants.

## Discussion

In the present study, we measured and compared the levels of three imprinted genes *CDKN1C*, *PHLDA2* and *IGF-2* in placental tissues between the SGA and AGA infants of full-term gestation. Upregulation of *CDKN1C* and *PHLDA2* as well as downregulation of *IGF-2* were detected at mRNA and protein levels in the SGA infants as compared with the AGA infants. Consistently, it has been reported that the mRNA levels of *PHLDA2* and *CDKN1C* were increased whereas the *IGF-2* mRNA levels were decreased in human intrauterine growth restriction (IUGR) placentas [12]. Nevertheless, the data from 36 full-term SGA and 41 full-term AGA infants did not show any associations of placental *PHLDA2* transcript abundances with maternal placental or neonatal parameters [18]. Moreover, upregulation of the *PHLDA2* mRNA levels were not detected in the SGA placentas [18]. This contradictory might be caused by the differences of the SGA samples. A combination of constitutionally small births and cases of fetal growth retardation were used. In addition, the SGA mothers were significantly shorter than the AGA ones; short, small mothers are genetically programmed to have smaller babies compared to their taller counterparts [18]. In chorionic villus samples that were collected at 12 weeks of gestation that subsequently resulted in normal live births at term, the SGA neonates had significantly lower *IGF-2* mRNA levels than the AGA ones [10]. Low *IGF-2* mRNA levels were also reported in the placentas in growth-restricted pregnancies [19,20]. Some studies assessed plasma *IGF-2* protein levels after birth in the SGA infants [21], showing downregulation in the full-term SGA infants than in the full-term AGA ones. Recently, a large birth cohort was performed to map the profile of imprinted genes in placental samples in large for gestational age (LGA), SGA and AGA infants [22]. Notably, early, term and late of gestational age were combined in this study [22]. 10 differentially expressed genes were identified across BW categories; 6 genes including *ABCA1*, *BLCAP*, *MEG3*, *MEST*, *NDN*, and *PLAGL1* were upregulated in the LGA infants compared to the SGA infants, and 4 genes including *DLK1*, *H19*, *IGF-2* and *NNAT* were upregulated in the LGA infants compared to the SGA and AGA infants [22].

In the present study, full-term infants were investigated. However, the PW of the SGA group was significantly lower than that of the AGA group. The Pearson’s coefficient analysis did not show significant correlation between PW and BW in the SGA infants, suggesting that placental factor may not have major influence on BW in our full-term SGA infants. In consistent with the previous findings [23,24], we found that placental *PHLDA2* protein levels were negatively associated with BW, particularly in the SGA infants. Similarly, in normal term babies, qPCR analysis shows that placental levels of *PHLDA2* but not *IGF-2* had a significant effect on BW, and that *PHLDA2* mRNA levels were negatively correlated with the size at birth [13]. Nevertheless, *PHLDA2* mRNA levels in 12 weeks chorionic villus samples did not correlated with BW [10]. Here, we revealed a positive correlation of *IGF-2* protein levels with BW only in the SGA infants. A statistically significant association between *IGF-2* mRNA levels in 12 weeks chorionic villus samples and BW was also observed [10]. In combination with our data, it suggests that maternally expressed *PHLDA2* is suppressing the baby’s growth later in pregnancy rather than early on, while the paternally expressed *IGF-2* is persistently promoting the baby’s growth since early stage of pregnancy. In addition, it has been shown that increased *CDKN1C* mRNA levels may be related to the increased percentage of the SGA births in ARTCP [16]. A higher negatively correlation of *CDKN1C* protein levels with BW was revealed in our SGA group compared to the AGA group. A large cohort study showed that a 2-fold increase in *MEST* expression was associated with decreased risk, while a 2-fold increase in *NNAT* expression was associated with increased risk for the SGA status [22]. Currently, the conclusions about the association of the expression levels of specific imprinted genes with BW are contradictory due to different conditions that were used. And anyhow, our findings suggest that the abundance of imprinted genes *CDKN1C*, *PHLDA2* and *IGF-2* in placental tissue had an important effect on fetal growth and the development of the full-term SGA infants.

All the three imprinted genes’ expression levels were altered in the placental tissue in full-term SGA infants. However, the relationship among the expression levels of *CDKN1C*, *PHLDA2* and *IGF-2* have not been reported. Our results only show a statistically positive correlation between *PHLDA2* and *CDKN1C* at the protein level in the SGA infants. The interaction of *PHLDA2* with *CDKN1C* and the underlying mechanisms should be further investigated.

SGA children undergo catch-up growth in early life, which mainly occurs from 6 months to 2 years after birth [1,25]. Approximately, 85% of SGA children have catch-up growth by age 2 years [1]. The mechanisms by which SGA infants and/or children develop catch-up growth remain unclear. The data from 50 full-term SGA children aged 12–18 months show that majority of SGA had catch-up growth by 18 months, and a good correlation with *IGF-1* levels was revealed [26]. In our study, body weight was monitored until 6 months after birth in full-term SGA infants. The weight of the SGA infants was significantly lower at all three time points 1, 3 and 6 months than that of the AGA infants. However, relative to BW, the weight gain of the SGA infants was statistically higher since 1 month after birth than that of the AGA counterparts. This demonstrates that the SGA infants underwent rapid catch-up growth compared to the AGA infants. Further analysis shows that *CDKN1C* and *PHLDA2* protein levels were positively correlated with catch-up growth, whereas *IGF-2* levels were negatively correlated with catch-up growth, at 1, 3 and 6 months in the SGA infants. Recently, it was reported that the proportions of overweight and obesity of the SGA children at 4 to 18 months after birth were significantly higher than those in the AGA children, with higher proportions in boys than in girls [27]. Given that abnormal BW, in particular, reduced BW, is implicated in metabolic syndrome including type 2 diabetes mellitus and cardiovascular disease, more studies should be performed to investigate the alterations and the pattern of the expression levels of imprinted genes since it has the potential to be developed as a novel biomarker for postnatal health outcomes. Additionally, intervention of alterations of placental imprinted genes might be a promising strategy to prevent SGA births and catch-up growth.

Taken together, our data demonstrated that increased *CDKN1C* and *PHLDA2* and reduced *IGF-2* levels in placental tissue were related to BW reduction and the catch-up growth of full-term SGA in early life. Placental *CDKN1C*, *PHLDA2* and *IGF-2* levels monitoring may be useful for prediction and prevention of the development of SGA births and its related postnatal metabolic diseases. Notably, the alterations of these imprinted genes’ expressions are likely a response, not a cause of reduced fetal growth in the SGA infants. Thus, it should be further investigated to prove the causative role of specific imprinted genes’ alterations in the development of SGA birth and its catch-up growth.
